# Reduced gray matter volume of the hippocampal tail in melancholic depression: evidence from an MRI study

**DOI:** 10.1186/s12888-024-05630-5

**Published:** 2024-03-05

**Authors:** Zhaosong Chu, Lijin Yuan, Kun Lian, Mengxin He, Yi Lu, Yuqi Cheng, Xiufeng Xu, Zonglin Shen

**Affiliations:** 1 Department of Psychiatry, The First Affiliated Hospital of Kunming Medical University, 650032 Kunming, China; 2 Yunnan Province Clinical Research Center for Mental Health, 650032 Kunming, China; 3 Department of Medical Imaging, The First Affiliated Hospital of Kunming Medical University, 650032 Kunming, China

**Keywords:** Melancholic depression, Hippocampus, Amygdala, Subregion, FreeSurfer

## Abstract

**Background:**

Melancholic depression (MD) is one of the most prevalent and severe subtypes of major depressive disorder (MDD). Previous studies have revealed inconsistent results regarding alterations in grey matter volume (GMV) of the hippocampus and amygdala of MD patients, possibly due to overlooking the complexity of their internal structure. The hippocampus and amygdala consist of multiple and functionally distinct subregions, and these subregions may play different roles in MD. This study aims to investigate the volumetric alterations of each subregion of the hippocampus and amygdala in patients with MD and non-melancholic depression (NMD).

**Methods:**

A total of 146 drug-naïve, first-episode MDD patients (72 with MD and 74 with NMD) and 81 gender-, age-, and education-matched healthy controls (HCs) were included in the study. All participants underwent magnetic resonance imaging (MRI) scans. The subregional segmentation of hippocampus and amygdala was performed using the FreeSurfer 6.0 software. The multivariate analysis of covariance (MANCOVA) was used to detect GMV differences of the hippocampal and amygdala subregions between three groups. Partial correlation analysis was conducted to explore the relationship between hippocampus or amygdala subfields and clinical characteristics in the MD group. Age, gender, years of education and intracranial volume (ICV) were included as covariates in both MANCOVA and partial correlation analyses.

**Results:**

Patients with MD exhibited a significantly lower GMV of the right hippocampal tail compared to HCs, which was uncorrelated with clinical characteristics of MD. No significant differences were observed among the three groups in overall and subregional GMV of amygdala.

**Conclusions:**

Our findings suggest that specific hippocampal subregions in MD patients are more susceptible to volumetric alterations than the entire hippocampus. The reduced right hippocampal tail may underlie the unique neuropathology of MD. Future longitudinal studies are required to better investigate the associations between reduced right hippocampal tail and the onset and progression of MD.

**Supplementary Information:**

The online version contains supplementary material available at 10.1186/s12888-024-05630-5.

## Introduction

Major depressive disorder (MDD) is a prevalent mental disorder characterized by persistent depressed mood, low energy, and diminished interest, with a lifetime prevalence in China estimated at 6.8% [1]. MDD is a highly heterogeneous psychiatric disorder with distinct subtypes based on different clinical features. One of the most common and severe subtypes of MDD is the melancholic depression (MD), characterized by anhedonia, psychomotor agitation, self-blame, early awakening, loss of appetite, and weight loss [2]. Previous studies have shown that MD accounts for more than half of all MDD cases [3, 4]. Compared to non-melancholic depression (NMD), MD patients tend to exhibit more severe clinical symptoms, experience more episodes of illness, have a higher risk of suicide, and demonstrate lower cognitive performance [5, 6]. Furthermore, the pathological mechanisms underlying MD differ from those of NMD, with distinctive hyperactivity of hypothalamic-pituitary-adrenal (HPA) activity [7], decreased levels of brain-derived neurotrophic factor (BDNF) [8], and alterations in reward circuit associated brain regions [9, 10]. These discrepancies in clinical presentation and biological indicators suggest that MD may have different pathological mechanisms. However, the exact neurobiological mechanisms of MD remain unclear.

With the rapid advancement in neuroimaging techniques and analysis methodologies, MRI has been increasingly used to study the mechanisms of MDD. Current evidence from neuroimaging studies suggests that dysfunction of the fronto-limbic system is intimately associated with the pathogenesis of depression [11]. The hippocampus and amygdala, as key components of the limbic system, play crucial roles in emotion processing, learning, and memory [12–15]. Changes in the structure or function of these regions are frequently related to MDD, characterized by symptoms such as depressed mood and poor cognitive function [16–20]. In addition, the midbrain-limbic dopaminergic reward pathway is a critical neurological basis for anhedonia, a core symptom of MD [21–23]. Therefore, the hippocampus and amygdala may be essential neural structural bases for MD. However, the grey matter volume (GMV) changes of the hippocampus and amygdala in MD have yielded inconsistent results, demonstrating unchanged GMV of the hippocampus [24, 25] and amygdala [24], smaller GMV of the hippocampus [26, 27], and larger GMV of the amygdala [25]. It is worth noting that these studies have considered the amygdala or hippocampus as a single, indivisible structure, ignoring the complexity of their internal structure.

Numerous animal and MRI studies show that the hippocampus and amygdala are composed of distinct subregions [28–30], each with different metabolite concentrations and functions [31]. For example, the cornu ammonis (CA)1 region of the hippocampus is associated with autobiographical memory [32] and self-awareness [33]; the CA3 region is associated with spatial working memory [34]; and the medial amygdala is associated with aggression [35]. Therefore, an increasing number of researchers have explored the structure and function of hippocampal or amygdala subregions using manual or automated segmentation techniques in mental disorders such as MDD [36], bipolar disorder [37], obsessive-compulsive disorder [38], and post-traumatic stress disorder [39]. This approach aims to reveal the specific roles of different hippocampal or amygdala subregions in these disorders.

In previous studies pertaining to depression, the CA1 volume has been demonstrated as a predictor of illness duration [40], while the hippocampal tail has shown associations with the efficacy of anti-depressant medications [41]. Furthermore, the volumes of various subregions within the hippocampus and amygdala have manifested correlations with the severity of depressive symptoms [42]. These findings collectively suggest a robust relationship between the subregional GMV of the amygdala and hippocampus and multiple clinical features of depression. However, there is presently only a single study focused on exploring dynamic functional connectivity pathways specifically within hippocampal subregions in MD [43]. A recent investigation, including 30 MDD patients with severe anhedonia, observed that these patients had reduced GMV of the CA1, granule cell and molecular layer of the dentate gyrus (GC-ML-DG), and molecular layer (ML) when compared to healthy individuals [44]. Nonetheless, it is important to note that anhedonia represents just one facet of the clinical features associated with MD. Consequently, this study did not elucidate the structural features of the hippocampal subregions in MD.

Thus, in the present study, we aimed to detect the volumetric alterations of each subregion of the hippocampus and amygdala in patients with MD and NMD to probe the neurobiological signature of these subtypes. In addition, we also sought to elucidate the association between clinical characteristics and the volume of specific subregions of the hippocampus and amygdala.

## Methods

### Participants

Between February 2012 and July 2015, we recruited 147 first-episode, drug-naïve, right-handed MDD patients aged 18 to 60 years from the outpatient clinic or inpatient wards of the Department of Psychiatry at the First Affiliated Hospital of Kunming Medical University. All patients met the DSM-IV diagnostic criteria for depression and scored at least 18 on the 17-item Hamilton Depression Rating Scale (HDRS). We excluded patients with other comorbid mental disorders, neurological illnesses, serious physical diseases, brain injury, substance abuse, pregnancy, or those who had received electroconvulsive therapy (ECT), transcranial magnetic stimulation (TMS), and systematic psychotherapy. Additionally, we recruited 81 healthy controls (HCs) through recruitment posters in local schools and communities. The HCs were matched to the MDD patients in age, gender, educational level, and handedness, and were free of psychiatric and neurological illnesses, serious physical diseases, substance abuse, pregnancy, and other contraindications to MRI scan.

All participants and their legal guardians signed the informed consent. This research was approved by the Ethics Review Board of Kunming Medical University, Kunming, Yunnan Province, People’s Republic of China.

### Melancholic features

The HDRS and Montgomery-Asberg Depression Rating Scale (MADRS) item scores were used to divide the MDD patients into the MD group and NMD group according to DSM criteria, as described in a previous study [45, 46]. In brief, the MD group met the following criteria: the MADRS item 8 ≥ 4 or MADRS item 1 or 2 ≥ 5 with at least three of the following symptoms: psychomotor disturbance (HDRS item 8 or 9 ≥ 1), guilt (HDRS item 2 ≥ 1), late insomnia (HDRS item 6 ≥ 1), and appetite/weight loss (HDRS item 12 or 16 = 2). Patients not meeting these criteria were categorized into the NMD group. In total, 72 MDD patients were assigned to the MD group, and 74 to the NMD group based on these criteria.

### Image acquisition

All subjects underwent an MRI scan before receiving antidepressant medication. MRI data were obtained on a Philips Achieva 3.0-T MRI scanner (Philips Healthcare, Best, The Netherlands) equipped with an eight-channel head coil. Restraining foam pads were used to minimize head motion during the scans. The T1-weighted MRI parameters were: TR/TE = 1900/20ms, slice thickness = 6 mm, FOV = 230 mm×190 mm, matrix size = 232 × 144, flip angle = 90°, axial slices = 18, scan duration time = 2min3s. The T2-weighted MRI parameters were: TR/TE = 2500/80ms, slice thickness = 6 mm, FOV = 250 mm×220 mm, matrix size = 332 × 225, flip angle = 90°, axial slices = 18, scan duration time = 55s. The three-dimensional (3D) volumetric structural MRI scan was performed after excluding structural abnormalities by T1 and T2-weighted MRI scans. The parameters of the 3D MRI scan sequence were: TR/TE = 7.4/3.4ms, slice thickness = 1.2 mm, FOV = 250 mm×250 mm, matrix size = 256 × 256, flip angle = 90°, slices = 230 with no gap, inversion time = 300ms, scan duration time = 6min53s.

### Subregions segmentation of hippocampus and amygdala

The reconstruction and segmentation of the entire and subregional hippocampus and amygdala were performed using the FreeSurfer 6.0 software (http://surfer.nmr.mgh.harvard.edu). Firstly, the image is preprocessed through the “recon-all” pipeline in Freesurfer [47]. The standard pipeline includes motion correction, skull stripping, Talairach transformation, intensity normalization, pial surface reconstruction, cortical and subcortical segmentation. Then, the hippocampal and amygdala subregional segmentation was performed using the automated algorithm provided in FreeSurfer. By automatic segmentation, each cerebral hemisphere’s hippocampus was subdivided into 12 subregions: parasubiculum, presubiculum, subiculum, CA1, CA3, CA4, GC-ML-DG, ML, hippocampus-amygdala transition area (HATA), fimbria, hippocampal tail, and hippocampal fissure. The amygdala was also divided into 9 subregions in each cerebral hemisphere, respectively, including the lateral, basal, accessory-basal, anterior-amygdaloid-area, central, medial, cortical, cortico-amygdaloid-transition, and paralaminar nucleus. Methods for segmenting the subregions of the hippocampus and amygdala have been detailed in depth in previous publications [48, 49]. Finally, volumetric data of the amygdala, hippocampus and their subregions, along with intracranial volume (ICV), were extracted for subsequent statistical analyses. The schematic of the segmentation of the hippocampal and amygdala subregions is presented in the Supplementary Fig. 1.

### Statistical analysis

All statistical analyses were performed using the Statistical Package for the Social Sciences (SPSS 25.0 for Windows). Two-sample t-test or analysis of variance (ANOVA) test was conducted to analyze differences in age, education, and clinical characteristics. The chi-square test was utilized for the analysis of gender difference. The significance threshold was set at *P* < 0.05. Controlling for age, sex, years of education, and ICV, a multivariate analysis of covariance (MANCOVA) was conducted by using a general linear model (GLM) to assess volumetric differences of the subregions of hippocampus and amygdala between groups. The Bonferroni correction was applied for multiple comparisons. A significance threshold of *P* < 0.0019 was set for hippocampus subregions analysis (*P* < 0.05/26, 24 subregions and 2 total volumes in bilateral hippocampus), while a significance threshold of *P* < 0.0025 was set for amygdala subregions analysis (*P* < 0.05/20, 18 subregions and 2 total volumes in bilateral amygdala). The partial correlation analysis was used to explore the relationship between abnormal hippocampus or amygdala subfields and clinical characteristics in the MD group, with age, sex, education level, and ICV controlled as covariates. The statistical significance threshold was set at *P* < 0.05 for this analysis.

## Results

### Demographic and clinical characteristics

No significant differences were observed among the NMD, MD, and HCs groups in terms of age, gender, education, BMI, ICV, and duration of illness (all *P* > 0.05). However, the total scores of MADRS and HDRS, and HDRS factor scores were significantly higher in the MD group compared to the NMD group, indicating that MD patients had more severe depressive symptoms than NMD patients (all *P* < 0.01) (Table 1).

**Table 1:** Demographic and clinical characteristics of MD, NMD, and HCs

Variable	MD (*n* = 72)	NMD (*n* = 74)	HCs (*n* = 81)	F/t/χ^2^	P
Sex (M/F)	16/56	26/48	25/56	3.035	0.219
Age (years)	35.49(11.12)	33.99(9.75)	35.25(11.56)	0.408	0.665
Education (years)	11.82(3.81)	11.20(4.67)	12.43(4.39)	1.571	0.210
Illness duration (months)	11.30(17 0.87)	12.07(14.58)		-0.283	0.777
BMI (kg/m^2^)	21.13(2.76)	20.94(2.85)	21.71(2.97)	1.537	0.217
ICV (mm^3^)	1290856.17(180328.07)	1344408.00(173137.61)	1325802.47(165573.73)	1.805	0.167
EHI-SF	98.44(7.55)	97.97(8.54)	98.61(9.27)	0.114	0.892
HDRS	26.10(4.83)	21.31(3.28)		6.986	< 0.001
MADRS	33.76(6.65)	25.85(5.21)		7.991	< 0.001
Anxiety/somatization factor	8.82(2.16)	7.88(1.74)		2.907	0.004
Weight factor	1.11(0.88)	0.35(0.67)		5.850	< 0.001
Cognitive dysfunction factor	4.42(1.85)	3.12(1.46)		4.682	< 0.001
Retardation factor	7.81(1.90)	6.74(2.14)		3.167	0.002
Sleep disorder factor	4.76(1.50)	3.70(1.86)		3.799	< 0.001

Abbreviations: MD, Melancholic depression; NMD, Non-melancholic depression; HCs, healthy controls; HDRS, Hamilton Depression Rating Scale; MADRS, Montgomery-Asberg Depression Rating Scale; ICV, Intracranial volume; EHI-SF, Edinburgh Handedness Inventory-short form; BMI, Body Mass Index

### Hippocampal volume differences among MD, NMD and HCs

**Fig. 1:**
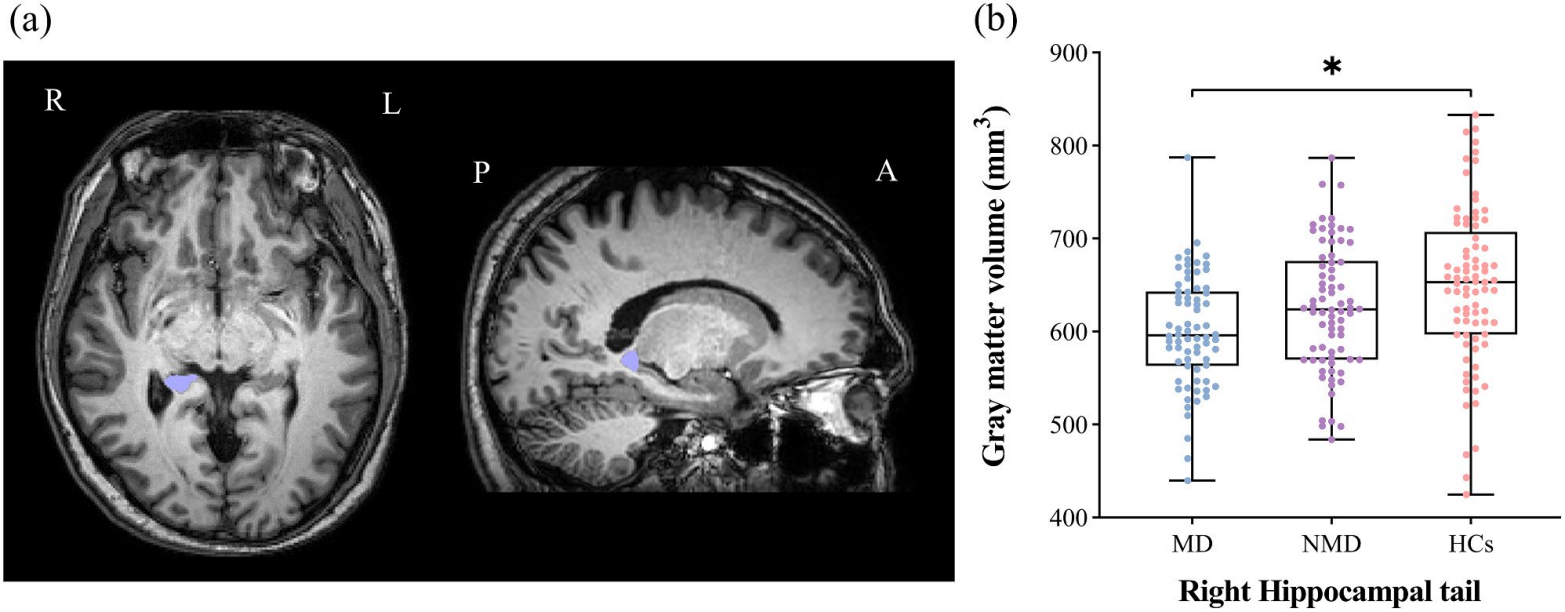
Group comparison of right hippocampal tail. (**a**) Schematic diagram of right hippocampal tail; (**b**) Group differences in the GMV of right hippocampal tail between MD, NMD, and HCs. *P-value: Bonferroni corrected. L, Left; R, Right; A, Anterior; P, Posterior; MD, Melancholic depression; NMD, Non-melancholic depression; HCs, Healthy controls

After adjusting for age, gender, education and ICV, the MANCOVA analysis revealed a significant difference in the GMV of the right hippocampal tail across the three groups (*P* < 0.001, Bonferroni corrected). *Post*-*hoc* analysis indicated that the GMV of the right hippocampal tail was significantly smaller in the MD group compared to HCs group (*P* < 0.001, Bonferroni corrected). However, after applying the Bonferroni-adjusted threshold, there were no statistically significant differences in the GMV of the right hippocampal tail between the NMD group and either the MD or HCs groups (Table 2; Fig. 1).

### Amygdala volume differences among MD, NMD and HCs

After adjusting for age, gender, education, and ICV, the MANCOVA analysis did not reveal any significant differences in the total or subregional gray matter volume (GMV) of the amygdala among the three groups, based on the Bonferroni-adjusted threshold (Table S1).

### Correlation analysis of altered subregion of hippocampus and clinical characteristics

In the MD group, the partial correlation analysis did not reveal any significant correlations between the GMV of the right hippocampal tail and illness duration (*r* = 0.036, *P* = 0.673), HDRS score (*r* = 0.121, *P* = 0.325), and MADRS score (*r* = 0.043, *P* = 0.727). Furthermore, there were no significant correlations between the GMV of the right hippocampus tail and the five factor scores of HDRS, including anxiety/somatization (*r* = 0.048, *P* = 0.700), weight (*r* = 0.067, *P* = 0.586), cognitive dysfunction (*r* = 0.137, *P* = 0.266), retardation (*r* = 0.221, *P* = 0.070), and sleep disorder (*r*= -0.097, *P* = 0.433), after controlling for gender, age, education, and ICV.

**Table 2:** Differences in GMV of the hippocampal subregions between MD, NMD, and HCs

Subregions	MD (*n* = 72)	NMD (*n* = 74)	HCs (*n* = 81)	F	P	Effect size (partial η^2^)	Post-hoc [P (95%CI)]		
							MD vs. NMD	MD vs. HCs	NMD vs. HCs
Left hemisphere									
Parasubiculum	62.52(11.31)	63.79(11.53)	64.50(11.89)	0.371	0.691	0.003	0.907(-3.382 to 3.809)	0.502(-4.666 to 2.294)	0.430(-4.890 to 2.091)
Presubiculum	310.00(34.09)	315.39(39.58)	317.50(46.66)	0.254	0.776	0.002	0.905(-10.932 to 12.343)	0.589(-14.357 to 8.172)	0.508(-15.095 to 7.498)
Subiculum	425.94(40.93)	437.71(49.07)	444.61(55.61)	1.993	0.139	0.018	0.603(-17.374 to 10.107)	0.056(-26.266 to 0.334)	0.169(-22.670 to 4.006)
CA1	610.84(61.98)	619.01(64.43)	624.93(68.58)	0.574	0.564	0.005	0.784(-16.092 to 21.307)	0.456(-24.959 to 11.241)	0.305(-27.619 to 8.685)
CA3	198.30(23.52)	200.08(25.70)	201.56(25.29)	0.153	0.858	0.001	0.873(-7.238 to 8.513)	0.709(-9.066 to 6.180)	0.592(-9.726 to 5.564)
CA4	242.47(21.64)	242.48(24.67)	245.91(24.87)	0.551	0.577	0.005	0.482(-4.632 to 9.784)	0.760(-8.057 to 5.895)	0.304(-10.654 to 3.339)
GC-ML-DG	282.80(24.70)	284.23(28.83)	287.35(28.70)	0.428	0.652	0.004	0.606(-5.997 to 10.259)	0.695(-9.435 to 6.299)	0.356(-11.589 to 4.191)
ML	544.84(45.16)	551.51(49.86)	560.57(54.31)	1.534	0.218	0.014	0.851(-12.669 to 15.335)	0.164(-23.166 to 3.940)	0.114(-24.538 to 2.646)
HATA	54.33(9.57)	54.98(8.50)	53.52(8.40)	0.500	0.607	0.005	0.711(-2.249 to 3.294)	0.325(-1.338 to 4.027)	0.548(-1.869 to 3.512)
Fimbria	80.28(16.80)	82.33(16.16)	81.56(14.72)	0.088	0.916	0.001	0.679(-3.607 to 5.525)	0.876(-4.069 to 4.770)	0.787(-5.041 to 3.823)
Tail	599.10(62.59)	607.27(77.67)	626.71(77.95)	2.347	0.098	0.021	0.931(-23.583 to 21.596)	0.058(-42.965 to 0.765)	0.072(-42.035 to 1.821)
Fissure	146.32(25.96)	147.05(29.82)	150.08(25.94)	0.361	0.697	0.003	0.732(-7.209 to 10.245)	0.624(-10.551 to 6.344)	0.400(-12.093 to 4.850)
Whole hippocampus	3411.43(260.91)	3458.79(306.12)	3508.71(331.67)	1.608	0.203	0.014	0.863(-73.677 to 87.794)	0.150(-135.361 to 20.930)	0.107(-142.645 to 14.096)
Right hemisphere									
Parasubiculum	60.95(8.75)	62.29(11.08)	60.91(10.17)	0.301	0.741	0.003	0.837(-3.469 to 2.812)	0.593(-2.214 to 3.866)	0.456(-1.894 to 4.203)
Presubiculum	292.00(34.62)	301.05(40.60)	297.60(38.52)	0.211	0.810	0.002	0.519(-14.612 to 7.398)	0.785(-12.126 to 9.179)	0.694(-8.549 to 12.816)
Subiculum	425.60(45.65)	440.44(49.66)	443.57(48.45)	1.730	0.180	0.015	0.294(-20.409 to 6.209)	0.065(-25.018 to 0.746)	0.443(-17.955 to 7.883)
CA1	660.47(68.13)	673.85(75.85)	682.20(82.83)	1.015	0.364	0.009	0.935(-22.426 to 20.643)	0.208(-34.195 to 7.492)	0.241(-33.363 to 8.444)
CA3	219.55(26.08)	225.79(28.24)	222.04(28.21)	0.241	0.786	0.002	0.535(-11.122 to 5.786)	0.955(-8.416 to 7.949)	0.559(-5.771 to 10.641)
CA4	253.08(24.09)	258.08(25.97)	256.38(26.80)	0.080	0.923	0.001	0.691(-9.173 to 6.087)	0.818(-8.248 to 6.522)	0.857(-6.726 to 8.087)
GC-ML-DG	294.87(27.90)	302.27(30.30)	300.27(31.78)	0.250	0.779	0.002	0.503(-11.801 to 5.803)	0.593(-10.835 to 6.204)	0.875(-7.861 to 9.227)
ML	571.30(51.31)	584.29(54.84)	588.70(61.05)	1.014	0.364	0.009	0.633(-19.265 to 11.75)	0.164(-25.647 to 4.373)	0.369(-21.933 to 8.174)
HATA	58.27(9.50)	58.27(9.58)	57.30(9.38)	0.463	0.630	0.004	0.627(-2.306 to 3.817)	0.337(-1.516 to 4.411)	0.647(-2.280 to 3.664)
Fimbria	75.95(14.74)	80.37(13.89)	78.78(15.74)	0.466	0.628	0.004	0.340(-6.450 to 2.236)	0.545(-5.497 to 2.909)	0.704(-3.402 to 5.029)
Tail	600.55(59.79)	625.55(67.67)	648.47(84.88)	7.659	< 0.001^*^	0.065	0.175(-35.920 to 6.568)	< 0.001^*^(-60.705 to -19.581)	0.016(-46.088 to -4.846)
Fissure	151.92(24.46)	162.03(34.13)	159.49(29.66)	1.643	0.196	0.015	0.085(-18.080 to 1.181)	0.177(-15.735 to 2.909)	0.668(-7.313 to 11.385)
Whole hippocampus	3512.59(294.61)	3612.26(322.89)	3636.24(368.49)	1.712	0.183	0.015	0.386(-127.254 to 49.412)	0.066(-165.671 to 5.328)	0.344(-126.997 to 44.494)

Notes: The data in the second, third and fourth columns of the table represent the mean volume (standard deviation), Unit: mm^3^. ^*^P-value: Bonferroni corrected. Abbreviations: MD, Melancholic depression; NMD, Non-melancholic depression; HCs, Healthy controls; CI, Confidence Interval; CA, cornu ammonis; GC-ML-DG, granule cell and molecular layer of the dentate gyrus; ML, molecular layer; HATA, hippocampus-amygdala transition area

## Discussion

Melancholic depression is a highly prevalent subtype of MDD, and its neurobiological basis involves aberrant changes in the structures and functions of specific brain regions [50, 51]. However, alterations in the morphology of the hippocampal and amygdala subregions in MD remain largely unknown. To the best of our knowledge, this is the first exploratory study aimed at detecting structural changes in hippocampal and amygdala subregions in patients with MD and NMD. Our most intriguing finding is that the right hippocampal tail was smaller in the MD group compared to the HCs group, a difference that reached statistical significance after applying the Bonferroni-adjusted threshold. Conversely, none of the other subregions of the hippocampus and amygdala, or their overall GMV, showed substantial differences among the MD, NMD, and HCs groups. Further analysis revealed that the change in right hippocampal tail volume was not significantly associated with clinical characteristics, such as the duration and severity of disease in the MD group.

The hippocampus is roughly divided into the hippocampal head, body and tail along the longitudinal axis of the hippocampus [30]. The hippocampal tail is attached to the hippocampal body and receives blood flow separately by the posterior hippocampal artery. Existing evidence suggests that the human hippocampal tail may play a role in processing spatial information and episodic memory [52, 53]. Therefore, our finding of reduced hippocampal tail GMV in MD may indicate dysfunction in processing spatial information and episodic memory in MD, which is consistent with previous studies [6]. In addition, the reduced hippocampal tail may lead to a disturbance of the midbrain-limbic dopaminergic reward system, which induces anhedonia in depression [21, 22]. Activation of postsynaptic 5-HT(1 A) receptors in the hippocampal tail has been reported to prevent learned helplessness [54], a key psychological mechanism that generates anhedonia in depression [55]. Thus, it is hypothesized that the reduced volume of the hippocampal tail in MD leads to reduced activation of its postsynaptic 5-HT(1 A) receptors, thereby triggering learned helplessness and ultimately anhedonia. Previous studies have found a positive correlation between hippocampal tail volume at baseline and antidepressant efficacy in MDD patients [27, 41, 56], indicating that hippocampal tail volume could be a biological marker for predicting the efficacy of antidepressant treatment. Although hippocampal tail volume did not differ significantly between the two subtypes of depression in our study, MD tended to have a smaller right hippocampal tail relative to NMD. This observation suggests a potential prediction that antidepressant drug efficacy may be worse in MD, and it aligns with findings from certain previous clinical studies [57, 58]. In line with previous research [24, 25], our findings revealed no discernable differences in GMV of the whole hippocampus between MD, NMD, and HCs. However, we did observe a statistically significant reduction in GMV of the right hippocampal tail in MD patients, suggesting that reduced right hippocampal tail may be a more sensitive indicator for identifying MD than whole hippocampal volume.

The amygdala, a crucial brain region within the limbic system anatomically linked to the hippocampus [30], has been reported to show covariation in volume alterations with the hippocampus in MDD [42]. Prior studies have yielded mixed results regarding changes in amygdala GMV in MDD, likely influenced by factors such as comorbidity, medication status, and the severity of illness [17, 20, 59]. In the meanwhile, the relationship between the amygdala GMV and MD remains unclear. For instance, although larger bilateral amygdala volumes have been reported in patients with MD relative to HCs [25], our study found no significant differences between the two groups. Upon further examination of amygdala subregions, we found no significant differences in GMV in amygdala subregions for MD compared to NMD and HCs. This implies that GMV changes within the amygdala and its subregions may not be associated with the melancholic features of depression.

Notably, our study did not identify any significant statistical differences in the total GMV of the hippocampus and amygdala or their respective subregions between individuals with MD and NMD. However, it cannot be briefly presumed that MD and NMD have identical structures within the hippocampus, amygdala, and their subregions. The categorization of MD and NMD was based on their scores on the HDRS and MADRS scales, which may have obscured potential differences between subtypes of depression within the NMD group. It is possible that NMD group includes various depression subtypes, such as those with anxious traits, atypical traits, or suicidal risk, which may explain the absence of observed differences in GMV between MD and NMD groups.

Our study does have certain limitations that need consideration. Firstly, as a cross-sectional study, we cannot establish a causal relationship between the development of MD and the structural changes in hippocampal and amygdala subregions. Moreover, while previous studies have demonstrated that hippocampal tail volume is associated with antidepressant treatment outcomes in MDD [41, 56], we were unable to explore the relationship between hippocampal tail volume and treatment effects in MD due to the cross-sectional design of our study. Thus, we plan to address these limitations in future longitudinal studies. Thirdly, previous studies found a close relationship between hippocampal tail and cognitive function [52, 53]. Unfortunately, we were not able to explore the correlation between hippocampal tail volume and cognitive function because cognitive function was not measured in subjects in this study. Finally, in our study, the categorization of MD and NMD relied on the HDRS and MADRS item scores instead of a structured questionnaire, which may have included other depressive subtypes within our NMD group, potentially affecting the accuracy of our results in NMD.

## Conclusions

In conclusion, our study provides evidence for a selective vulnerability of hippocampal subregions in MD, particularly in the right hippocampal tail. Our findings highlight the need of future research on MDD to examine microstructural changes within the hippocampus and amygdala regions. Such investigations are crucial for us to gain a better understanding of the underlying mechanisms of MD and to develop more effective interventions.

### Electronic supplementary material

Below is the link to the electronic supplementary material.

**Supplementary Material 1:** The schematic of the segmentation of the hippocampal and amygdala subregions

**Supplementary Material 2:** Differences in GMV of the amygdala subregions between MD, NMD, and HCs

## Data Availability

The datasets used and/or analyzed during the current study available from the corresponding author on reasonable request.
