# Supplementary figures and images for: Reduced gray matter volume of the hippocampal tail in melancholic depression: evidence from an MRI study
Source: BMC Psychiatry. 2024 Mar 5;24:183. doi: 10.1186/s12888-024-05630-5 (PMC10913289; doi:10.1186/s12888-024-05630-5)

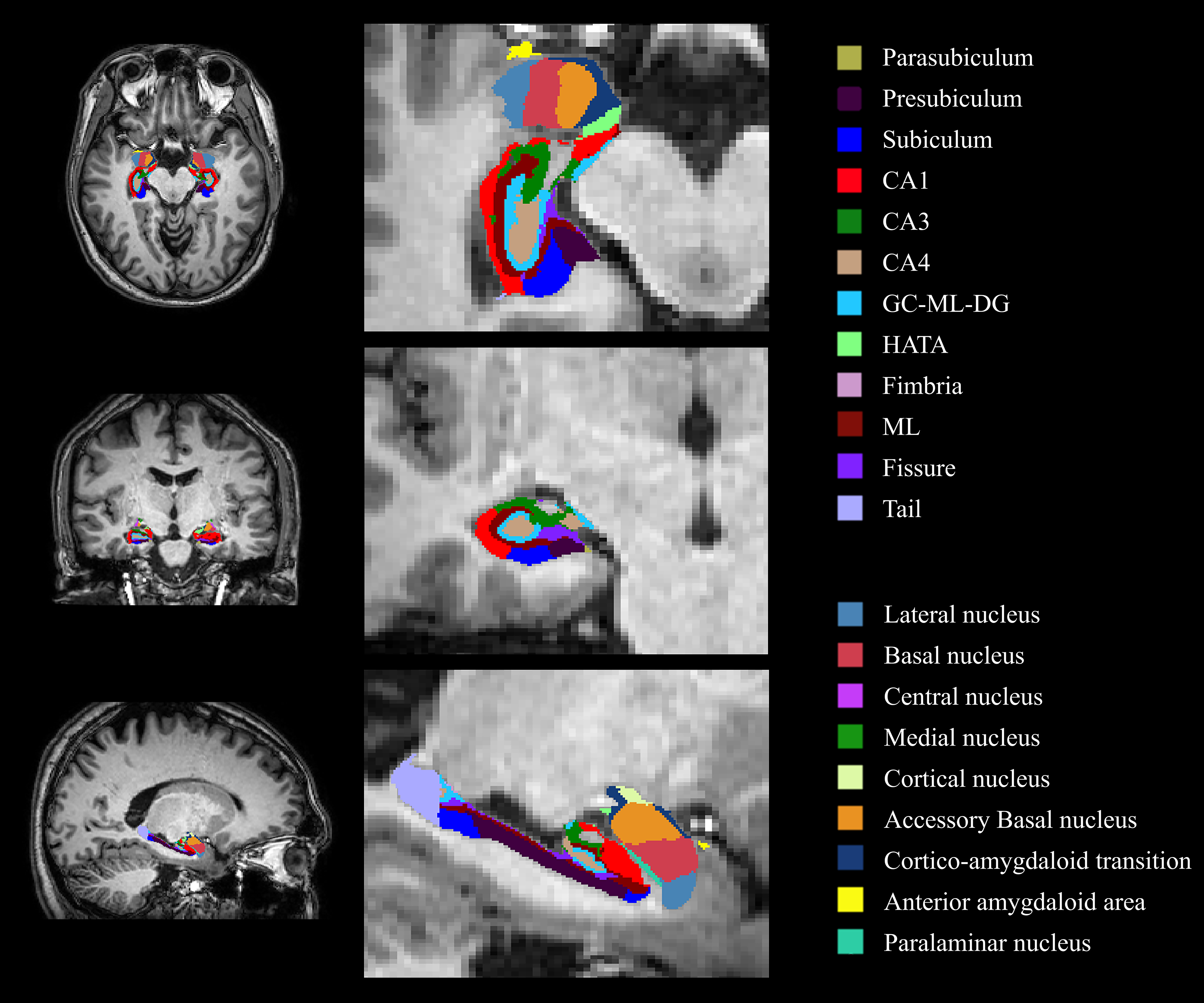

Supplement: Supplementary file 1 — Supplementary Material 1: The schematic of the segmentation of the hippocampal and amygdala subregions [file 12888_2024_5630_MOESM1_ESM.tif]
