# Supplementary material for: Reduced gray matter volume of the hippocampal tail in melancholic depression: evidence from an MRI study
Source: BMC Psychiatry. 2024 Mar 5;24:183. doi: 10.1186/s12888-024-05630-5 (PMC10913289; doi:10.1186/s12888-024-05630-5)
Supplement: Supplementary file 2 — Supplementary Material 2: Differences in GMV of the amygdala subregions between MD, NMD, and HCs [file 12888_2024_5630_MOESM2_ESM.docx]

**Table S1** Differences in GMV of the amygdala subregions between MD, NMD, and HCs

| **Subregions** | **MD (n=72)** | **NMD (n=74)** | **HCs (n=81)** | ***F*** | ***P*** | **Effect size**  **(partial *η^2^*)** | ***Post-hoc* [*P* (95%*CI*)]** | | |
| --- | --- | --- | --- | --- | --- | --- | --- | --- | --- |
|  |  |  |  |  |  |  | **MD vs. NMD** | **MD vs. HCs** | **NMD vs. HCs** |
| Left hemisphere |  |  |  |  |  |  |  |  |  |
| Lateral nucleus | 630.62(78.46) | 642.35(76.39) | 635.46(62.39) | 0.206 | 0.814 | 0.002 | 0.616(-13.369 to 22.499) | 0.548(-12.064 to 22.654) | 0.934(-16.679 to 18.139) |
| Basal nucleus | 423.05(53.84) | 429.77(54.85) | 431.63(44.06) | 0.525 | 0.592 | 0.005 | 0.501(-8.766 to 17.891) | 0.753(-14.963 to 10.839) | 0.314(-19.563 to 6.314) |
| Accessory Basal nucleus | 257.43(31.39) | 254.58(31.07) | 260.34(30.15) | 2.391 | 0.094 | 0.021 | 0.051(-0.053 to 17.146) | 0.863(-7.596 to 9.051) | 0.066(-16.166 to 0.529) |
| Anterior amygdaloid area | 55.84(8.22) | 56.31(8.10 | 56.24(7.07) | 0.435 | 0.648 | 0.004 | 0.355(-1.168 to 3.245) | 0.695(-1.710 to 2.561) | 0.573(-2.755 to 1.529) |
| Central nucleus | 42.74(7.65) | 42.22(8.34) | 44.81(9.12) | 2.601 | 0.077 | 0.023 | 0.258(-1.114 to 4.135) | 0.266(-3.977 to 1.104) | 0.024(-5.495 to -0.400) |
| Medial nucleus | 24.98(7.05) | 24.16(6.60) | 26.93(7.65) | 3.205 | 0.042 | 0.028 | 0.251(-0.954 to 3.632) | 0.180(-3.733 to 0.707) | 0.012(-5.078 to -0.626) |
| Cortical nucleus | 27.56(4.70) | 26.39(4.73) | 27.72(4.72) | 3.567 | 0.030 | 0.031 | 0.014(0.365 to 3.203) | 0.678(-1.084 to 1.664) | 0.034(-2.872 to -0.117) |
| Cortico-amygdaloid transition | 177.98(22.44) | 178.09(21.44) | 176.22(22.10) | 1.173 | 0.311 | 0.011 | 0.195(-2.120 to 10.327) | 0.174(-1.857 to 10.191) | 0.983(-5.978 to 6.105) |
| Paralaminar nucleus | 48.49(6.59) | 49.47(6.40) | 49.16(5.49) | 0.123 | 0.884 | 0.001 | 0.631(-1.213 to 1.995) | 0.888(-1.442 to 1.663) | 0.723(-1.838 to 1.277) |
| Whole amygdala | 1688.7(199.34) | 1703.34(195.16) | 1708.51(167.87) | 0.759 | 0.469 | 0.007 | 0.244(-19.178 to 74.861) | 0.795(-39.506 to 51.516) | 0.347(-67.479 to 23.805) |
| Right hemisphere |  |  |  |  |  |  |  |  |  |
| Lateral nucleus | 654.8(76.58) | 676.85(72.92) | 662.39(65.77) | 0.537 | 0.585 | 0.005 | 0.468(-24.790 to 11.426) | 0.795(-15.217 to 19.837) | 0.314(-8.586 to 26.57) |
| Basal nucleus | 442.56(54.19) | 453.17(47.67) | 447.04(50.12) | 0.092 | 0.912 | 0.001 | 0.993(-13.211 to 13.092) | 0.717(-10.383 to 15.076) | 0.711(-10.361 to 15.172) |
| Accessory Basal nucleus | 271.38(33.64) | 274.55(28.57) | 276.68(34.25) | 0.322 | 0.725 | 0.003 | 0.611(-6.608 to 11.218) | 0.788(-9.808 to 7.447) | 0.428(-12.138 to 5.166) |
| Anterior amygdaloid area | 60.65(9.34) | 61.39(7.25) | 60.01(8.49) | 0.607 | 0.546 | 0.005 | 0.635(-1.910 to 3.125) | 0.274(-1.080 to 3.794) | 0.547(-1.695 to 3.193) |
| Central nucleus | 44.82(9.63) | 44.54(7.61) | 47.09(9.36) | 1.901 | 0.152 | 0.017 | 0.405(-1.578 to 3.891) | 0.280(-4.103 to 1.191) | 0.054(-5.267 to 0.042) |
| Medial nucleus | 25.17(8.02) | 25.37(7.53) | 26.11(7.60) | 0.191 | 0.826 | 0.002 | 0.859(-2.271 to 2.723) | 0.678(-2.928 to 1.907) | 0.550(-3.161 to 1.688) |
| Cortical nucleus | 28.59(4.55) | 28.84(4.25) | 29.41(4.92) | 0.564 | 0.570 | 0.005 | 0.612(-1.024 to 1.736) | 0.591(-1.701 to 0.970) | 0.290(-2.060 to 0.618) |
| Cortico-amygdaloid transition | 186.39(20.75) | 189.2(21.40) | 183.92(22.28) | 1.220 | 0.297 | 0.011 | 0.853(-5.734 to 6.929) | 0.155(-1.693 to 10.563) | 0.220(-2.309 to 9.983) |
| Paralaminar nucleus | 48.47(5.85) | 50.14(6.16) | 49.03(5.39) | 0.429 | 0.652 | 0.004 | 0.548(-1.927 to 1.026) | 0.768(-1.215 to 1.644) | 0.362(-0.768 to 2.098) |
| Whole amygdala | 1762.83(200.52) | 1804.05(172.99) | 1781.70(187.04) | 0.085 | 0.919 | 0.001 | 0.936(-49.662 to 45.780) | 0.761(-39.040 to 53.341) | 0.699(-37.232 to 55.415) |

Notes: The data in the second, third and fourth columns of the table represent the mean volume (standard deviation), Unit: mm3. Abbreviations: MD, Melancholic depression; NMD, Non-melancholic depression; HCs, Healthy controls
